# Reading digital- versus print-easy texts: a study with university students who prefer digital sources

**DOI:** 10.1186/s41155-022-00212-4

**Published:** 2022-05-06

**Authors:** Noemí Bresó-Grancha, María José Jorques-Infante, Carmen Moret-Tatay

**Affiliations:** 1 Escuela de Doctorado, Universidad Católica de Valencia San Vicente Mártir, San Agustín 3, Esc. A, Entresuelo 1, 46002 València, Spain; 2 MEB lab, Faculty of Psychology|, Universidad Católica de Valencia San Vicente Mártir, Avenida de la Ilustración, 4, Burjassot, Valencia, 46100 Spain; 3 Dipartimento di Neuroscienze Salute Mentale e Organi di Senso (NESMOS), La Sapienza University of Rome, Rome, Italy

**Keywords:** Reading, Print, Digital, Cognition load, Shallowing hypothesis, Comprehension, Word recognition

## Abstract

The transition from on-paper to on-screen reading seems to make it necessary to raise some considerations, as a greater attentional effort has been claimed for print texts than digital ones. Not surprisingly, most university students prefer this digital medium. This research aims to examine reading times by contextualizing this phenomenon into two processes: namely, word recognition and reading comprehension task on paper and on screen. Thus, two different tasks—counterbalanced into digital and print mediums—were carried out per each participant with a preference for a digital medium: a reading comprehension task (RCT) and a lexical decision task (LDT) after reading a specific story. Participants were slower reading print texts and no statistically significant differences were found in RCT accuracy. This result suggests that the task required more cognitive resources under the print medium for those with a worse comprehension performance in reading, and a more conservative pattern in digital RCT for those with a better performance.

## Introduction

Reading has different effects on the brain, is considered as an activity that can reduce stress (Corazon et al., 2010), improves people’s memories (Peng et al., 2018), and enhances empathic skills (Gabay, Dundas, Plaut, & Behrmann, 2017; Kuzmičová, Mangen, Støle, & Begnum, 2017; Mangen, 2016), among others. This process has also been linked to longer life spans (Chang, Wu, & Hsiung, 2020; Peng et al., 2018), but, surprisingly for most, it is not innate. It must be learned through specific exercises for this purpose, usually in our childhood. In this way, networks of connections are developed through an architecture which is already used for recognizing visual patterns and understanding spoken language (Vogel et al., 2013). This architecture has been mainly addressed by studying response times (RTs) (Luce, 1986) and the specificity of reading-related regions through fMRI (Vogel et al., 2013).

When describing the reading process, an abstraction is developed in the fusiform gyrus that allows our brain to recognize strings of letters in milliseconds. This occurs even if stimuli are presented in different typographies, sizes, or even upper or lower cases, among others (Perea, Moret-Tatay, & Panadero, 2011). The changing nature and circumstances of reading, as digitization is growing, is a subject of debate that might influence our reading process. Literature has suggested a greater attentional effort for print texts than for digital texts (Mangen & Kuiken, 2014). While some studies stipulate higher reading comprehension on paper (Kim & Kim, 2013; Mangen, Walgermo, & Brønnick, 2013), others find no such differences between mediums (Porion, Aparicio, Megalakaki, Robert, & Baccino, 2016; Rockinson- Szapkiw, Courduff, Carter, & Bennett, 2013). These differences on results might be explained through the effect of modulating variables (Delgado, Vargas, Ackerman, & Salmerón, 2018) such as time constraints, genre or type of text, and temporal moment. Moreover, a piece of research (Mangen, Olivier, & Velay, 2019) stands out by assessing this variable in Kindle DX, as a digital medium, and print. Participants were assessed in their reconstruction of the story from both mediums. The authors concluded that kinesthetic feedback was less informative in a Kindle. Another study comparing print, e-reader, and a tablet computer by combining EEG and eye-tracking measures showed shorter mean fixation durations and lower EEG theta band voltage density in the digital medium for older participants (Kretzschmar et al., 2013). However, comprehension accuracy did not differ between mediums.

Comparisons in this front have also been addressed from fields such as ergonomics and perception (Benedetto, Drai-Zerbib, Pedrotti, Tissier, & Baccino, 2013, 2014). It should be noted that the physical interaction that occurs while reading on paper or on screen is significantly different. Actions like turning a page or feeling the paper of a book produce a multisensory experience that increases the cognitive, affective, and emotional insertion in the subject matter (Jacobs, 2015; Kuzmičová et al., 2017; Mangen et al., 2019). In this context, a shallowing hypothesis is theorized, which tries to explain how recent media technologies might lead to a decline in reflective thought and an increase in superficial learning, as an immediate reward is expected (Annisette & Lafreniere, 2017). Given this change in pattern, could decoding be different from one medium to the other, being more superficial in digital, and thus affecting comprehension?

Classical authors such as Eldredge (1988) claimed that repeated exposure to frequently words in print mediums is likely to improve learners’ visual recognition of those words, which in turn is also likely to improve reading comprehension. Not surprisingly, this is an accepted strategy in fluency interventions (Brown et al., 2018). One of the main classical models on text comprehension defines this process as the result of the interaction of text features and readers' knowledge, involving variables such as propositional representation and readers' prior knowledge (Hsu, Clariana, Schloss, & Li, 2019; McNamara & Kintsch, 1996). In this way, familiarity with the medium is a variable of interest, and differences can be stipulated between samples that are more familiar with the digital versus print environment. On the other hand, considering the simple view of reading (Hoover & Gough, 1990) as one of the most influential approaches which has been developed to address early reading comprehension, reading must be addressed from a cognitive perspective. More precisely, this cognitive model of reading comprehension (RC) stipulates that RC is a consequence of the interaction between decoding and linguistic comprehension, where word recognition can be considered part of the decoding process (de Oliveira, da Silva, Dias, Seabra, & Macedo, 2014; Kirby & Savage, 2008).

According to the literature, the most commonly employed cognitive tasks involved in printed word identification have been lexical decision (LDT) and naming (Imbir et al., 2020; Katz et al., 2012; Navarro-Pardo, Navarro-Prados, Gamermann, & Moret-Tatay, 2013), bearing in mind that in both tasks RTs and accuracy are the main dependent variables. Moreover, one of the dependent variables that RC and lexical decision tasks have in common is the analysis of reaction time or response latency. Within this field, processing speed has been described as an indicator of reading performance in groups such as participants with dyslexia (Norton & Wolf, 2012), and is considered a reflection of brain architecture (Luce, 1986; Moret-Tatay, Gamermann, Navarro-Pardo, de Córdoba, & Castellá, 2018). Some studies seem to indicate that readers in digital media spend less time than in printed texts; however, their understanding may also be affected (Ackerman & Lauterman, 2012). Therefore, the literature has stipulated that readers may overestimate their understanding of digital texts.

According to the literature, a decline of long-form reading in higher education is diminishing (Baron & Mangen, 2021). In this way, university students seem to prefer, or more frequently used, digital sources than print ones for short times of reading (Terra, 2015). Differences between mediums, which were previously described, might be of interest for to be addressed, particularly for short texts. For this reason, this research aims to examine the differences between digital and print easy-texts reading in University students who prefer digital sources. We hypothesize that university students with a preference for digital texts have lower reading latency for simple digital texts than print ones. In addition, we expect, in this profile of participants, shorter lexical decision latencies in digital versus print easy-texts. If comprehension is not compromised in easy texts, previous literature might reflect a cost optimization, which becomes more controversial in long texts, as previous literature has shown. A RC and word recognition across mediums were selected for this research question. Moreover, if word recognition is considered directly linked to RC, it is expected a similar pattern for both processes in terms of speed processing. Lastly, a Bayesian approach was considered as an alternative strategy to support traditional analysis as described in prior literature (Nuzzo, 2014; Puga, Krzywinski, & Altman, 2015).

## Materials and methods

### Participants

A sample of 40 university students (25 women and 15 men with an average age of 18.90 years and SD= 1.51), with no history or evidence of neurological or psychiatric disease, volunteered to participate. All participants were Spanish native speakers, without any neurological disorder reported and normal or corrected vision, and a preference for digital texts than print ones. All participants indicated a higher use of digital media than print, although specific usage was not quantified. They were randomly divided into two counterbalanced groups to perform the experiment tasks. In this way, one subgroup first performed the digital task and after that the print one, while the other way around was employed for the second subgroup. The tasks were carried out in accordance with the Declaration of Helsinki and approved by the University ethical committee. The ethical code is UCV/2020-2021/030. Participants gave written consent to participate in the study.

### Materials

A total of two tasks were carried out per participant: a reading comprehension task (RCT) and a lexical decision task (LDT).

For the reading comprehension task, two stories published in previous literature (Perea, Panadero, Moret-Tatay, & Gómez, 2012) were selected with its correspondent reading comprehension questions. These two stories have been successfully employed with children and employed in a counterbalanced design as the one carried out in the original research. In other words, the participants were divided into two groups, one read one of the stories in digital and the other one in print, while conversely in the other group. In terms of presentation, the whole text was presented in a single screen or in a single piece of paper. One text has a total of 153 Spanish words and the second one 162, with the title “The Wind” and “The Snowman” (see Perea et al., 2012). Afterwards, participants had to write in a piece of paper the answer to five questions related to each text, which were the same questions employed in the original study.

The second part of the session was dedicated to carry out the LDT. The stimuli consisted of a set of 120 five-letter words in Spanish and 120 pseudowords. Words were divided into two lists (to enable the comparison between print and digital in a counterbalanced way) and matched in terms of frequency and orthographic neighbors, as in the original publication (Moret-Tatay & Perea, 2011)^1^. It should be noted that this material was also originally developed for children and published in previous research work, but it has been employed in other experiments with other Spanish groups that differ in age, such as university and senior students (Navarro-Pardo et al., 2013; Perea, Devis, Marcet, & Gomez, 2016). By using these materials employed in children and older adults, the reading of simple texts is to be examined. Moreover, current results might allow future comparisons in different age groups.

All stimuli were presented with a fixed text that was the same for the digital and printed versions, in lowercase 14 pt Times New Roman. The viewing distance was optional for the participants, who were free to approach the text as closely as they wished, but with a maximum distance of 30 cm. For ecological validity reasons, the chosen presentation of texts was carried out as follows: the digital texts were presented on the computer screen, while the printed ones were presented on a sheet of paper on the table.

### Procedure

As previous mentioned, participants had to perform a LDT and a RCT. Thus, two lists of words and two versions for each story, one for a digital and one for a print medium, were prepared and randomly counterbalanced across participants. Half of the participants were initially presented with one text and word list, (e.g., digital), and then the other text and word list (e.g., on paper). The participants were tested in a quiet room at the University structure in small groups. The presentation of the stimuli and recorded response times were controlled by computers through the Windows software DMDX (Forster and Forster, 2003).

**Fig. 1:**
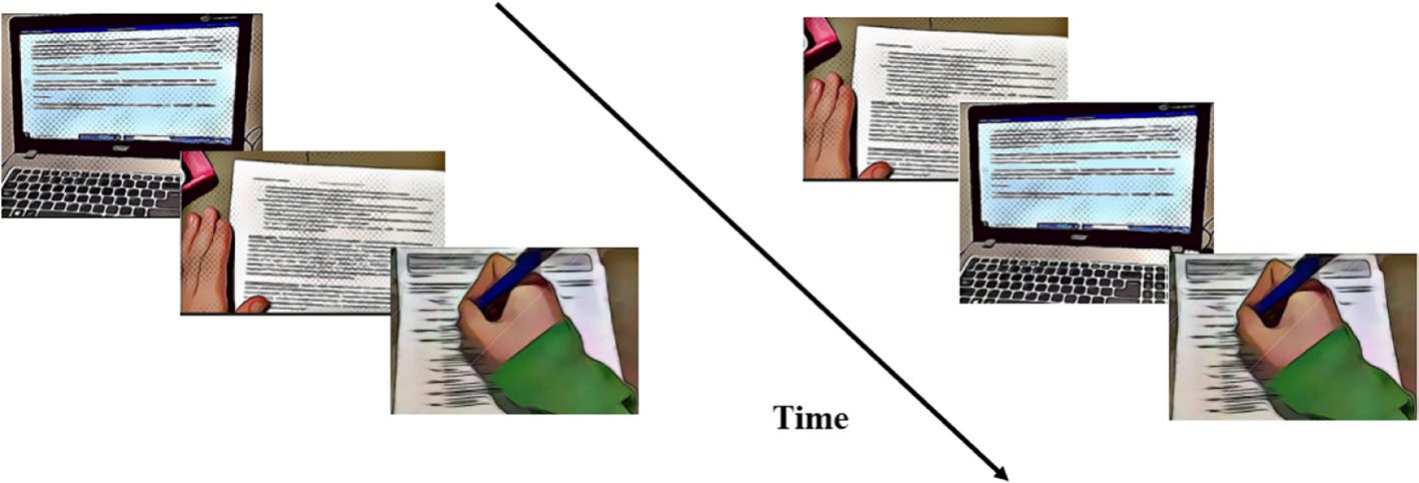
RCT task. The task was counterbalanced in the order of presentation of reading material according to media type: digital or print (left versus right). Finally, written comprehension questions were asked in written form

The presentation of the stories was counterbalanced across digital and print media, as shown in Fig. 1. It should be noted that for the RCT there was no maximum time in both print and digital mediums, the time employed for each participant was recorded. Participants were encouraged to employ the time they needed to properly read the text. They must press a key in the keyboard to indicate the starting and the same bottom to indicate the reading end in both mediums, similar than the LDT procedure. After the reading phase, they were asked to answer five questions out of time in a paper medium. Therefore, the dependent variables used were the unrestricted reading times (paper versus digital) and accuracy was measured.

**Fig. 2:**
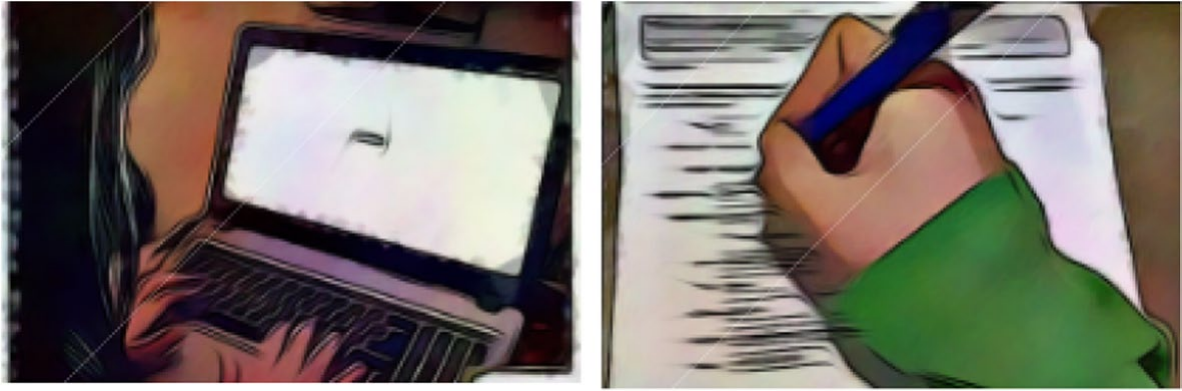
The classical LDT. The left-hand page is the paper adaptation for the LDT where participants crossed the words from pseudowords

For the LDT digital version, after pressing a starting bottom, a fixation point (+) was presented for 500 ms in the center of the screen. Then the target stimulus was presented until the participant’s response, with a maximum of 2500 ms. Participants were instructed to press a button (labeled “yes”) if the stimulus corresponded to an existing word in Spanish, and press another button (labeled “no”) if it did not. As depicted in Fig. 2, for the paper-based adaptation of the TDL, randomized lists of words were generated on printed paper, and participants were challenged to cross out the words against the pseudo-words.

In this case, participants had to press a button in DMDX to start and finish the task, and their timing was compared with the digital version. For the whole experiment, participants were instructed to respond as quickly as possible, maintaining a reasonable level of accuracy. The stimuli were randomly presented to each participant. Each session lasted about 20 minutes. Since reaction time might vary slightly between words and pseudo-words into each medium (due to obvious differences in media manipulation), only the accuracy between digital and print word recognition was taken as the dependent variable.

### Data analysis

Sample size was estimated with G*Power software (Faul, Erdfelder, Lang, & Buchner, 2007). A non-parametric approach was carried out, as some assumptions, such as normality, were not accomplished on the RCT times and LDT accuracy of the participants. Correlation was used to examine how performance in RCT and word recognition were related to each other. Furthermore, a Bayesian inference was carried out using the Bayes factor notation (BF10), as a strategy that offers several benefits from frequentist statistics in terms of Interval confidence for parameters, as well as evidential trajectory in favor of H_1_ over H_0_ (Marsman & Wagenmakers, 2017). This approach also allows to quantify uncertainty about effect sizes more easily (Moret-Tatay, Wester, & Gamermann, 2020). It should be noted that that measure of interest, the RTs, can be non-normally distributed, so other approaches might be of interest to shed light on the barriers to reach assumptions such as data normality. In this way, the Bayes factor notation (BF10) was employed to support H_1_ over H_0_ where, according to medium theories, word recognition and reading times predict comprehension, and differences might appear depending on the format. Data analysis was performed by using JASP (Version 0.12.2) [Computer software].

## Results

Descriptive analyses on the variables of interest are depicted in Table 1. It should be noted that reading time in the RCT was remarkably longer for on-paper reading than for on-screen reading. The same pattern was identified in reading comprehension and word recognition tasks. A test of normality (Shapiro-Wilks) was conducted on the variables under study. Statistically significant results (all *p*<.01) suggested that these variables were not normally distributed. For this reason, a non-parametric approach was carried out.

**Table 1:** Descriptive analysis on the three variables of interest: comprehension (over 5 points), word recognition accuracy (over 1 point), and reading time in milliseconds

Task	Variable	Print		Digital	
		Mean	SD	Mean	SD
RCT	Accuracy	3.63	1.08	3.45	1.13
	Time (ms)	71115.84	25987.32	39712.30	11107.19
LDT	Accuracy	.98	.02	.97	.02

*SD* standard deviation

A Wilcoxon signed-ranks test indicated that reading time in a print medium was higher than in a digital one, and the rank-biserial correlation (*r*) between both measures was employed as a measure for effect size in this non-parametric approach: *Z*=− 5.05; *p*<.001; *r*=.91; 95% CI [.84, .96]. This difference was also statistically significant in LDT accuracy; however, the confidence interval did not support this difference: *Z*=− 3.84; *p*<.001; *r*= − .04; 95% CI [− .37, .30]. RCT accuracy also reached the statistical significance level: *Z*= − 1.16; *p*<.001; *r*= − .50; 95% CI [− .72, -.20]. Table 2 depicts the Spearman correlations across variables, showing a strong correlation between RCT accuracy of digital and print media (rho=0.608; *p*<0.01). Moreover, RCT time in digital media correlated with RCT accuracy in digital in a positive way (rho=0.39; *p*<0.05), but not in print media. RCT time in print inversely correlated with accuracy in both digital (rho= − 0.339; *p*<0.05) and print mediums (rho= − 0.263; *p* <0.01). Finally, digital LDT only correlated with RCT time (rho=0.334; *p* <0.05), while print LDT correlated with both print (rho=0.319; *p*<0.05) and digital reading time (rho=0.335; *p*<0.05).

**Table 2:** Spearman’s rho correlations on the three variables of interest: comprehension (over 5 points), word recognition accuracy (over 1 point), and reading time in milliseconds

	1	2	3	4	5	6
Accuracy RCT in print (1)	—					
Accuracy RCT in digital (2)	0.608***	—				
Time RCT in print (3)	− 0.263**	− 0.339*	—			
Time RCT in digital (4)	0.175	0.392*	− 0.130	—		
Accuracy LDT in print (5)	0.319*	0.335*	− 0.162	0.129	—	
Accuracy LDT in digital (6)	0.150	0.334*	0.235	0.043	0.415*	—

* *p* < .05, ** *p* < .01, *** *p* < .001

**Fig. 3:**
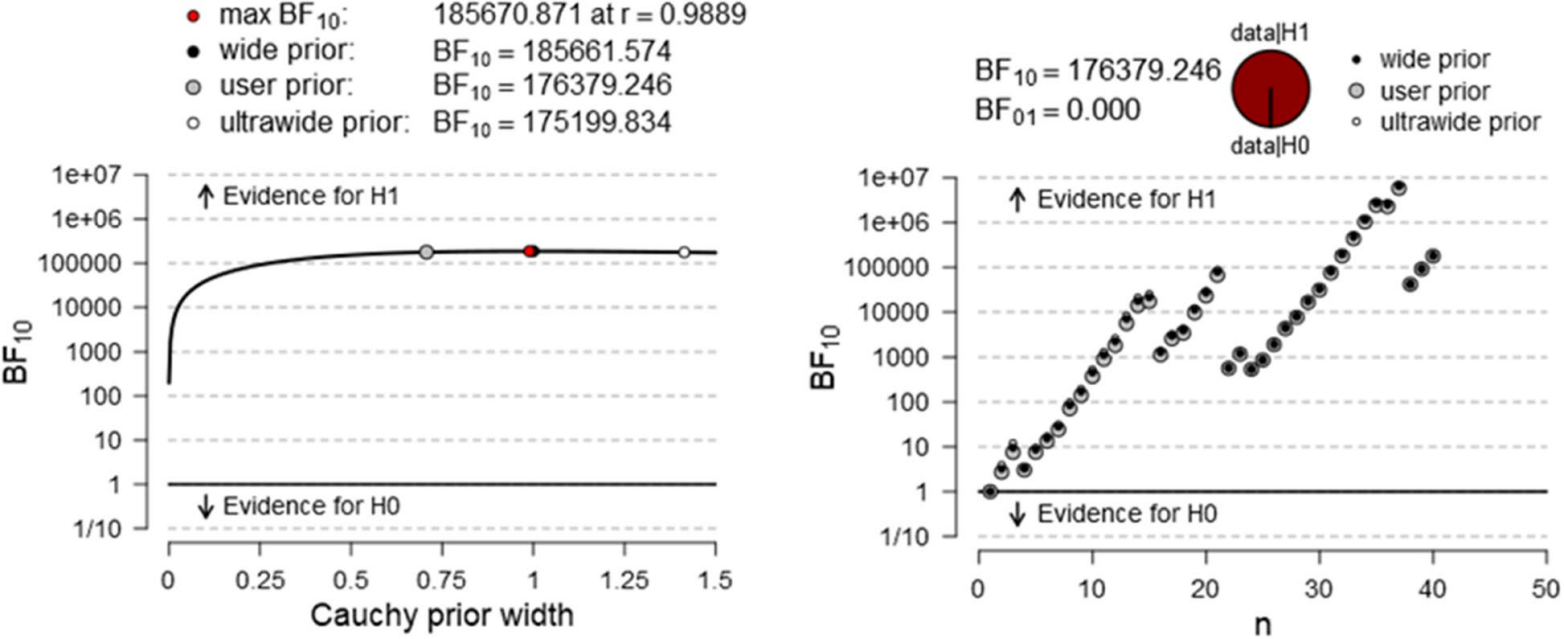
Bayes factor robustness check for differences between RCT time on screen and paper format

**Fig. 4:**
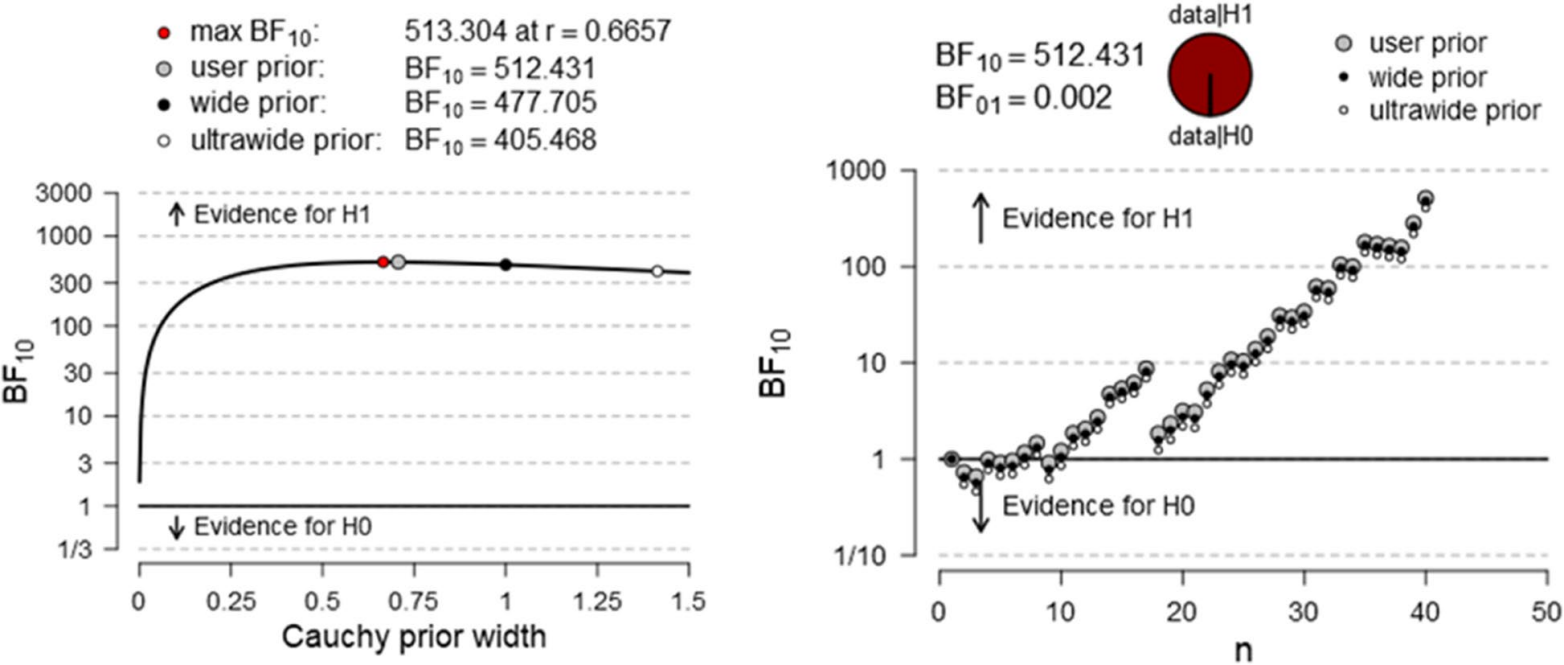
Bayes factor robustness check for differences between LDT accuracy on digital and paper support

**Fig. 5:**
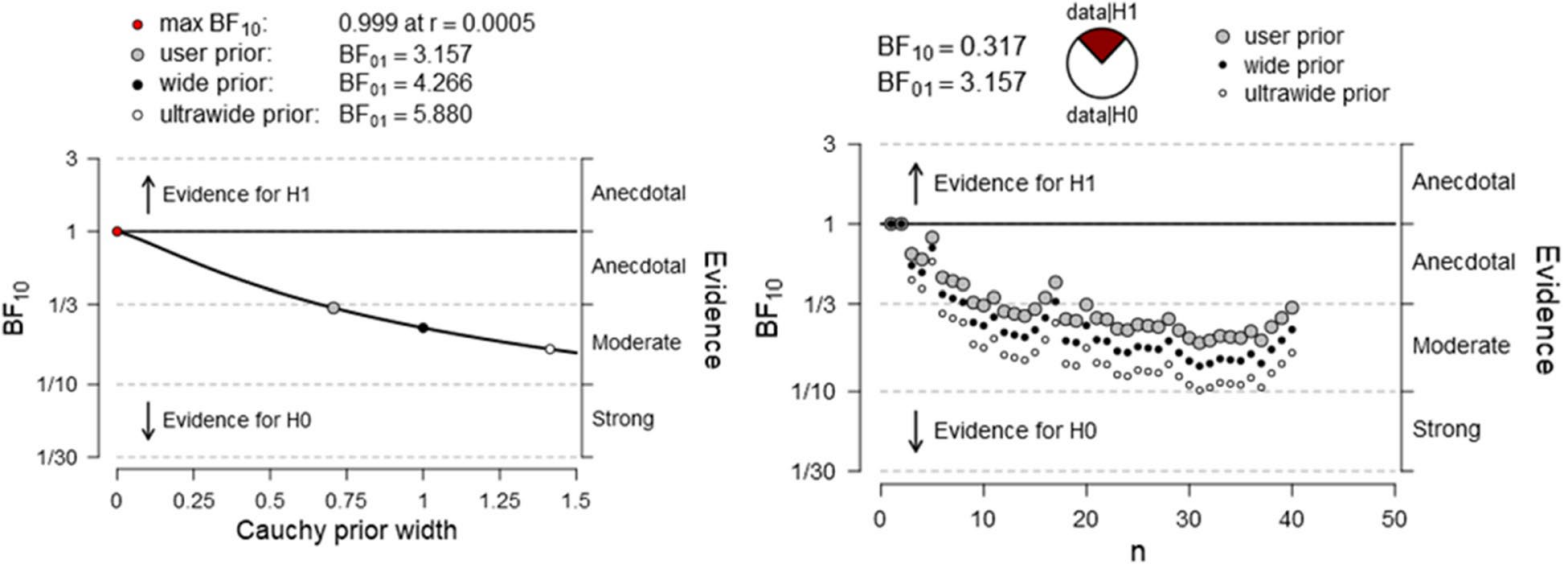
Bayes factor robustness check for differences between RCT accuracy on digital and paper support

A Bayesian paired procedure was also carried out across mediums. Figures 3, 4, and 5 illustrate the evidential trajectory in favor of H_1_ over H_0_. It must be pointed out that the evidence for the alternative hypothesis is relatively stable for reading times and word recognition, thereby suggesting that the analysis is robust. However, the evidence in favor of H1 in RC is not anecdotical.

## Discussion

The digital proliferation has attracted the scientific community’s interest towards the reading process in the last decade (Clinton, 2019). A switch towards superficial from deep reading processes has been described in digital mediums (Mangen & Kuiken, 2014), also suggesting that those who often read in print texts are less likely to perform multiple tasks during reading than those who often read in digital screens. Not surprisingly, it has been claimed that this difference is related to encouraging divided attention in this last medium (Sanbonmatsu, Strayer, Medeiros-Ward, & Watson, 2013). Even if detrimental effects have been reported regarding digital texts, university students seem to prefer digital sources (Terra, 2015). Even if mixed results have been found in the literature on long texts, what are the differences between reading digital- versus print-easy texts in university students who prefer the digital medium? Do these differences influence individual word recognition or only general comprehension?

The current results show a longer reading time for printed texts compared to digital texts. From the Bayesian approach, it is interesting to note that the most robust variables on the differences between print and digital texts were RCT time and LDT accuracy, but not for RCT accuracy. A feasible explanation related to RCT time would be related to the fact that printed texts allow readers to see and feel the spatial extent and physical dimensions of the text. In this way, the number of stimuli in printed texts would be enlarged, ultimately providing a greater number of physical, tactile, and spatio-temporal cues for reading, even for easy texts. However, comprehension seems not to be affected under such a level of demand. This might also explain higher attentional levels. However, the current manipulation does not allow to claim this explanation, as attentional levels were not measured, but are of interest for future lines of research. What these results did attempt to measure was the level of comprehension, which although slightly higher in the printed format, it was not conclusive. This result could support that young people would adapt to this form of reading as opposed to the older adult groups described in previous literature with techniques such as EEG (Kretzschmar et al., 2013). However, although the results seem promising, replications with other age groups are necessary to establish such e-generation claim.

The second main aim of the study was to address the relationship between RC and LDT accuracy in both mediums under study. An inverse relationship is found between RCT time and accuracy for print. However, this pattern changes for digital texts. This could reflect a familiarization cost from one medium to another. On the other hand, the direct relationship between RCT accuracy and time in digital texts might reflect a more conservative pattern of response in this medium that is more familiar to participants. Finally, a relationship was found between RCT and LDT accuracy per each medium. RCT accuracy in digital was also related to LDT accuracy in print, but not inversely. This would mark differences in individual patterns, as marked in previous literature regarding how text is integrated between mediums (Latini, Bråten, & Salmerón, 2020). Nevertheless, it must be considered that the results were not direct, suggesting that more variables underlie this process. One variable of interest—and it was not controlled in the current study—is the motivation during the task. This is one of the main limitations of this study. Moreover, the task employed here could be very easy for university students. This material was employed to avoid any “floor effect” and allows future comparison with other age groups.

It should be noted that being faster in the reading task has been also linked to the search for immediate reward in previous literature (Ackerman & Lauterman, 2012; Delgado et al., 2018). If so, that might explain shorter RTs under the digital, which in this case, might not affect comprehension in an easier task as the one under study. Despite the well-known preference for digital texts, regardless of the task and the benefits offered by the medium (for example, portability and speed), there are certain controversies regarding them, particularly considering groups of readers with dyslexia, who showed a more effective and comprehensive reading in digital texts (Chen & Keong, 2017; Schneps, Thomson, Chen, Sonnert, & Pomplun, 2013), but in these cases, the difficulty of the text was not controlled.

Finally, we would like to highlight the extra value of an integrative strategy such as the Bayesian approach in this field (Moret-Tatay, Beneyto-Arrojo, Laborde-Bois, Martínez-Rubio, & Senent-Capuz, 2016; Nuzzo, 2014; Ruiz-Ruano, López-Puga, & Delgado-Morán, 2019), by offering additional evidence to support differences between digital and print materials. In this regard, the Bayesian correlation depicted not particularly strong H_1_ in some cases. To sum up, the results of this study seem not to ensure the superiority of electronic texts over print ones in the prediction of reading time, as latency is not a single indicator, and as superficial pattern seems to be depicted even for easier texts.

## Conclusions

The aim of this study was to examine the relationship between RCT accuracy, RCT time, and LDT accuracy in print and digital mediums in university students with a preference for digital texts. For this reason, two experiments were carried out into a counterbalanced order across digital and print environments. Results can be described as follows: (i) latencies for reading on digital texts were shorter in digital than print mediums; (ii) Word recognition and Comprehension were slightly higher for print than digital texts, but not conclusive (iii) Reading time on digital and print text strongly correlated to each other; (iv) A relationship regarding LDT accuracy in print and RCT accuracy in print and digital texts was found, but this is not the case with LDT accuracy in digital.

## Footnotes

1 As in the original research by Moret-Tatay and Perea et al. (2011) the same 120 words from this research were employed, 60 were of high frequency (mean = 146.7 per million, range = 30.9–675.6) and 60 low frequency (mean = 10.2 per million, range = 0.7–23.2). Moreover, and as previously mentioned, the same two lists of stimuli than in the original research were employed, with 60 words and 60 nonwords being randomly assigned to List 1 and the other 60 words and 60 nonwords being assigned to List 2. In this way, those participants who were initially were presented with List 1 in Block 1, the second block would be List 2 and vice versa.
